# Supplementary material for: Sleep problems and chronic conditions in single parents in Ghana: Serial mediating roles of health-related quality of life and functional limitations
Source: PLoS One. 2024 Dec 31;19(12):e0312312. doi: 10.1371/journal.pone.0312312 (PMC11687747; doi:10.1371/journal.pone.0312312)
Supplement: Attachment — Submitted filename: Response to Reviewers.docx [file pone.0312312.s002.docx]

**Reviewer comments**

**Reviewer #1:**

This study is relevant and was presented in an intelligible fashion. The introduction was engaging, and set a strong foundation for the article. The conclusion and implications also tie back to the main points and offer insights for future research.

**Response:** We thank the Reviewer for the compliment. We take note of this positive comment with much gratitude, particularly regarding the intelligible nature of our study.

**Reviewer #2:**

The findings of this study can inform public health policies to improve sleep hygiene in countries where single parenthood is prevalent. I suggest some comments for improved readability of the paper.

**Response:** We appreciate the observation that our study can inform public health policies to improve sleep hygiene among this vulnerable population. We would like to thank the reviewers for a nice summary of our study and for revealing the potential interest of our paper in contributing to the academic and policymaking communities. We have taken note of all the constructive comments provided and have attempted to improve the quality of the paper further, guided by these critical comments..

Title

The title seems to suggest that single parenthood is the variable of interest in this study, which is not the case. In fact, single parents are the study population. Please change the title to reflect the study design.

**Response:** Thank you for this crucial observation. The title has been revised as follows;

“Sleep problems and chronic conditions in single parents in Ghana: Serial mediating roles of health-related quality of life and functional limitations.”

Abstract

Please do not introduce an acronym without spelling the full term at its first appearance.

**Response:** Acronyms such as low—and middle-income countries (LMICs) and health-related quality of life (HRQoL) have been spelled out fully in their first use. Please see Page 2 of the abstract.

“No study has reported the serial mediation effects of physiological factors in this association.” I am not sure if I would categorize HRQoL and functional limitations as physiological factors.

**Response:** Thank you for this. Physiological factors have been replaced with functional limitations and poor health-related quality of life. Please see page 2 of the abstract.

“The hypothesized association”? Where are the hypotheses?

**Response:** We have stated two hypotheses in the introduction section of the revised paper (see page 5). We have indicated that:

1) We hypothesized that there would be significant positive association between sleep problems and chronic conditions

2) We further hypothesized that the positive association between sleep problems and chronic conditions would be serially mediated by functional limitations and poor health-related quality of life.

**Methods**

Is the dependent variable the number of chronic conditions? Please explain clearly.

**Response:** The dependent variable is chronic conditions, which consist of 11 chronic physical conditions. Please see page 8.

Why did the authors use the sum of EQ-5D scores instead of the EQ-5D index, which can be calculated using the value set and is commonly used as a continuous variable?

I am not sure if you can sum EQ-5D responses (1, 2, to 3) and use the sum as a continuous variable to conduct Pearson's correlation and OLS. That is because EQ-5D responses are not interval scale variables.

**Response:** We truly understand the reviewer's drift in this regard. We would like to humbly say that, like the EQ-5D-5L, the EQ-5D-3L has been standardized and emanated a continuous index that has been used to perform linear regressions and Pearson’s correlations in many previous studies. The credibility of such an index has been demonstrated.

Lines 138-139. If 6 items are measured on 1 or 2, the total score should range from 6 to 12. Please check the accuracy of the statement.

**Response:** Thank you for this observation. We scored the responses of each of the six items on a binary (no=1 or yes=2) scale. This means that the total minimum score will range from 6 to 12, as you rightly indicated. We have revised and included this in the revised manuscript. Please see page 9.

**Discussion**

This section is a bit wordy. For example, can you summarize the review of the literature (Line 243-262), which seems a bit lengthy? In general, streamlining the discussion would enhance readability.

**Response:** We have summarized the literature review in the discussion section. Thank you very much. The summary is done on page 14.

**Specific comments**

Line 83, administered, is duplicative

**Response:** This has been rectified on page 6.

Line 108, what does the acronym SSA stand for?

**Response:** The acronym SSA stands for sub-Saharan Africa.

Line 153, the first mention of the acronym should be explained.

**Response:** The acronyms NHIS and SPSS stand for National Health Insurance Scheme and Statistical Package for Social Scientists, respectively. We have defined these fully in their first use.

Line 166, Cis  Cis

**Response:** It now reads confidence intervals (CIs) on page 10

Line 173, confidence intervals  Cis

**Response:** It is now consistent as confidence intervals (CI). Please see page 10.

Line 182, “gainfully employed,” sounds like a colloquial expression. Can you just use “employed”?

**Response:** Thank you for this correction. This has been done on page 10.

Line 185, can you add the unit for physical activity?

**Response:** The unit for physical activity has been added as 330.79 MET min

Line 231, what do you mean by “in a dose-response manner”?

**Response:** “a dose-response manner” has been explained as showing the magnitude of sleep problems' effect on chronic conditions. Please see page 13.

Line 324, check the reference style.

**Response:** It has been done on page 16.

We are grateful for your time spent reviewing and offering essential suggestions and additions that have significantly improved our paper. We also thank you for giving us another opportunity to revise and resubmit it to *Plos One*.

Yours sincerely,

(Corresponding author)
